# Progress of the Unique Fellowship in Health Research Evidence Synthesis in Nepal

**DOI:** 10.3126/nje.v15i4.88535

**Published:** 2025-12-31

**Authors:** Anju Vaidya, Padam Simkhada, Ram Chandra Silwal, Priya Paudyal, Meghnath Dhimal, Bibha Simkhada, Edwin van Teijlingen

**Affiliations:** 1 School of Human & Health Sciences, University of Huddersfield, Huddersfield, UK; 2 Green Tara Nepal, Kathmandu, Nepal; 3 Institute for Global Health & Wellbeing School of Medicine, Keele University, Keele, UK; 4 Nepal Health Research Council, Kathmandu, Nepal; 5 Faculty of Health & Medical Sciences, Bournemouth University, Bournemouth, UK

Nepalese researchers, academics, policymakers and practitioners are undertaking a unique Fellowship in evidence synthesis and evidence-based policy making as we highlighted in the June 2025 editorial in *Nepal Journal of Epidemiology* [1]. This Fellowship is part of a larger project called ‘Evidence-Informed Health Policy Making in Nepal (EHPN)’, funded by The British Council. Evidence-informed policy making developed out of the earlier idea of ‘evidence-based policy making’. The central idea behind evidence-based policy making was that it should be largely (or even solely) guided by evidence. Evidence-informed policy making adds that policies should not just be evidence-based they should also be feasible, appropriate for their context and aligned with stakeholders’ values and therefore requiring input meaningful input from stakeholders.

Our interdisciplinary team advertised the Fellowship through professional channels and social media to recruit highly motivated participants in Nepal. Those who applied went through a rigorous selection process. There were close to one hundred applicants, and we believe that there would have been additional applicants, had it not been for the social media ban and the fragile political context in Nepal during the recruitment phase. The selection criteria included: (a) academic achievement (Master’s degree and above) with some experience of academic writing/publication; and (b) approval from their respective organisations. During the selection process, we ensured that we recruit diverse range of participants in terms of gender, career stage and organisational background, including government and private universities and institutions.

The capacity-strengthening programme for our fellows will use an interactive approach and include a combination of in-person sessions with several practical examples, complemented with online sessions both before and after the in-person training. The curriculum comprises 16 sessions, starting with on introduction to evidence-informed policy making (EIPM) to developing an effective policy brief (see Table 1). The curriculum includes the traditional elements of systematic reviewing [2-3], whilst adding new elements such as constructing and producing a policy brief [4].

As part of the EHPN, the team offers mentorship to each of the Fellows. Mentors are experts in different topic areas, systematic reviewing, evidence synthesis, and/or research methods. All mentors are volunteers from Nepal, the UK and various other countries, including the USA and Qatar. Through such mentorship, the participants undertaking the fellowship will not only gain critical knowledge in evidence synthesis but also around academic writing and dissemination while also benefiting from networking opportunities (e.g., co-authorships opportunities with other fellow and mentors) that expand their professional horizons and future collaborations. It is also expected that the participants will publish their work by the end of the fellowship programme, thereby increasing its visibility within the wider scientific community and enhancing their career progression.

Each of the Fellows was signed two tutors for their own work. In addition, each Fellow also became part of a small learning cohort with another Fellow, this was not only to ensure that each Fellow had a person to act as second reviewer or second data extractor, but also to motivate each other. With both Fellows working in each other’s project would also qualify them as potential co-authors on each other publications. This mentorship element is longer term and continues after the formal Fellowship training as finished.

In summary, through the promotion of shared expertise and strengthening local capacity in evidence synthesis, this unique Fellowship project will ensure a robust mechanism for long-term sustainability of an evidence-synthesis centre in Nepal, beyond the project duration.

## Figures and Tables

**Table 1:** Fellowship Programme Overview of 16 Sessions

Session	Delivery	Activities
**1**	Online	Introduction to Fellowship Programme and EIPM
**2**	Online	Topic selection & Formulation of Review Questions
**3**	Face-to-face	Defining Inclusion/Exclusion Criteria & Develop Searched
**4**	Face-to-face	Practice Session: Database Search
**5**	Face-to-face	Protocol Development
**6**	Face-to-face	Study Selection
**7**	Face-to-face	Data Extraction
**8**	Face-to-face	Quality Assessment
**9**	Face-to-face	Approaches to Qualitative Synthesis
**10**	Face-to-face	Approaches to Quantitative Synthesis
**11**	Face-to-face	Practical Session: Data Synthesis
**12**	Online	Writing for Publication
**13**	Face-to-face	Understanding Policymaking & Identifying Opportunities for Influence
**14**	Face-to-face	Turning Evidence into Action
**15**	Online	Writing Effective Policy Briefs
**16**	Online	From Submission to Publication: Navigating Reviews & Proofs
